# Silicon regulation of manganese homeostasis in plants: mechanisms and future prospective

**DOI:** 10.3389/fpls.2024.1465513

**Published:** 2024-12-05

**Authors:** Yuebu Hailai, Yuan Liu, Zhengming Yang, Ying Li, Jingqiu Feng, Wenbing Li, Huachun Sheng

**Affiliations:** ^1^ Institute of Qinghai-Tibetan Plateau, Southwest Minzu University, Chengdu, Sichuan, China; ^2^ Sichuan Provincial Qiang-Yi Medicinal Resources Protection and Utilization Technology and Engineering Laboratory, Southwest Minzu University, Chengdu, Sichuan, China; ^3^ Tibetan Plateau Ethnic Medicinal Resources Protection and Utilization Key Laboratory of National Ethnic Affairs Commission of the People’s Republic of China, Southwest Minzu University, Chengdu, Sichuan, China

**Keywords:** beneficial element, Mn disorder, Mn bioavailability, subcellular distribution, scavenging ROS

## Abstract

Manganese (Mn), a plant micronutrient element, is an important component of metalloprotein involved in multiple metabolic processes, such as photosynthesis and scavenging reactive oxygen species (ROS). Its disorder (deficiency or excess) affects the Mn-dependent metabolic processes and subsequent growth and development of plants. The beneficial element of Si has a variety of applications in agricultural fields for plant adaptation to various environmental stresses, including Mn disorder. The probable mechanisms for Si alleviation of Mn toxicity in plants are summarized as follows: (1) Si alters the rhizosphere acidification, root exudates and microorganisms to decrease the bioavailability of Mn in the rhizosphere; (2) Si down-regulates Mn transporter gene and reinforces the apoplastic barriers for inhibiting the Mn uptake and translocation; and (3) Si promotes the Mn deposition onto cell wall and Mn compartmentation into vacuole. Under Mn-deficient conditions, the probable mechanisms for Si promotion of Mn absorption in some plants remain an open question. Moreover, scavenging ROS is a common mechanism for Si alleviating Mn disorder. This minireview highlights the current understanding and future perspectives of Si regulation of manganese homeostasis in plants.

## 1 Introduction

Manganese (Mn) is an essential element for plants, which is an integral part of the oxygen-evolving complex (OEC) of photosystem II (PSII) and serves as a cofactor for more than 30 enzymes, including Mn superoxide dismutase (MnSOD) and oxalate oxidase (Alejandro et al., 2020). Regardless of plant species, Mn should be accumulated at least 30 mg kg^−1^ dry weight in tissues to satisfy the demand for optimal growth and development (Broadley et al., 2012). If the accumulation of Mn in plants is below 10-20 mg kg^−1^ dry weight, Mn deficiency will occur (Broadley et al., 2012). Mn deficiency causes a lower net photosynthesis rate because the Mn-limited PSII is unstable and the development of chloroplast is inhibited (Schmidt et al., 2016). Under severe Mn-deficient conditions, leaves of plants will display brownish or necrotic spots in the tips, attributing to a decrease in MnSOD activity and thus their chloroplast impaired by the increased free oxygen radicals (Broadley et al., 2012; Hajiboland, 2012).

In contrast, high Mn causes a variety of symptoms in plants, and various plant species and genotypes have varying harmful Mn amounts (Fernando and Lynch, 2015). Generally, chlorotic leaves and necrotic spots are the most common symptoms of Mn toxicity among plant species (Millaleo et al., 2010), accompanied by decreased net photosynthetic efficiency and chlorophyll content (Alejandro et al., 2020). It is also observed that the uptake and translocation of other essential elements such as calcium (Ca), magnesium (Mg), iron (Fe), and phosphorus (P) are prevented in the Mn-stressed plants (Blamey et al., 2015; Lešková et al., 2017). Taken together, the maintenance of Mn homeostasis is required for normal plant growth and development.

The metalloid of silicon (Si) is classified as a quasi-essential element because of its proven protective and beneficial effects during plant adaptation to the environmental stresses (Coskun et al., 2019; Mandlik et al., 2020; de Tombeur et al., 2023). Si can be absorbed and translocated by Si transporters in plants only in the form of monomeric silicic acid (H_4_SiO_4_) (Mitani-Ueno and Ma, 2021), which is an uncharged molecule in solutions with a pH below 9. After H_4_SiO_4_ enters the plant body, it will deposit as inorganic hydrated SiO_2_ by silicification and form an organosilicon by covalent crosslinking with cell wall components (Liang et al., 2015; Sheng and Chen, 2020). It was proposed that plants silicify with a role in alleviating the nutritional imbalances (Pavlovic et al., 2021), such as the regulation of C: N: P homeostasis (Costa et al., 2024) and mitigation of boron disorder (Sheng et al., 2024), as well as the alleviation of Mn deficiency and toxicity (Che et al., 2016; Oliveira et al., 2021, 2022). To date, Si alleviation of Mn disorder-induced symptoms has been reported in many plants, including rice, cucumber, sorghum, cowpea, bean, maize, barley, sunflower, sugarcane, and others (**Table 1**). In this minireview, we focus on the underlying mechanisms and future perspectives of Si regulation of Mn homeostasis in plants.

**Table 1:** Si alleviation of Mn disorder in many plants.

Species	Mn concentration	Si supply	Symptoms	Si effects	Proposed mechanisms	References
*Oryza sativa* L. cv. Xinxiangyou 640	2 mM	Root fertilization (1.5 mM)	Plant growth inhibition; Chloroplast degradation	Improved plant growth; Decreases toxic symptoms	Stabilizing the structure of PSI and up-regulating the expression of photosynthesis-associated genes; Regulating Mn transport and antioxidant reactions	Li et al., 2012, 2015
*Oryza sativa* L. cv. Oochikara	200 μM	Root fertilization (1.0 mM)	Shoot growth inhibition; Brown spots in the old leaves	Improved plant growth; Decreases toxic symptoms	Down-regulating the expression of Mn transporter gene; Inhibiting the Mn uptake and root-to-shoot translocation	Che et al., 2016
*Cucumis sativus* L. cv. Chinese long	100 μM	Root fertilization (1.5 mM)	Plant growth inhibition; Brown spots; Small chlorotic regions with necrosis	Improved plant growth; Decreases toxic symptoms	Modulating the metabolism and utilization of phenolic compounds; Decreasing hydroxyl radical accumulation in the leaf apoplast	Dragišic´ Maksimovic´ et al., 2007, 2012
*Zea mays* L. cv. Kneja 605	200 or 500 μM	Root fertilization (1.0 mM)	Plant growth inhibition; Chloroplast damage	Improved plant growth; Decreases toxic symptoms;	Increasing the thickness of the epidermal layers; Accumulating the callose	Doncheva et al., 2009
*Sorghum bicolor* L.	0 μM	Root fertilization (1.0 mM) or leaf spraying (1.0 g/L)	Grain production drawdown; Shoot growth inhibition	Improved grain production; Decreases deficient symptoms	Enhancing antioxidant system and Mn use efficiency	de Oliveira et al., 2019
*Vigna unguiculata* L.	50 μM	Root fertilization (1.44 mM)	Brown spots	Decreases toxic symptoms	Promoting the Mn binding to the cell walls; Maintaining the reduced state of the apoplast	Iwasaki et al., 2002a, b
*Phaseolus vulgaris* L.	100 or 1000 ppm	Root fertilization (0.75 or 40 ppm)	Plant growth inhibition	Improved plant growth	Mediating the Mn compartmentation into the vacuole	Horst and Marschner, 1978
*Helianthus annuus* L.	400 μM	Root fertilization (1.4 mM)	Trichomes blackening	Decreases toxic symptoms	Being co-located with the Mn (Formation of Si-Mn complex)	Blamey et al., 2018; Van der Ent et al., 2020
*Glycine max* L.	30 μM	Root fertilization (1.4 mM)	Small chlorotic regions with necrosis	Decreases toxic symptoms	Being co-located with the Mn (Formation of Si-Mn complex)	Blamey et al., 2018; Van der Ent et al., 2020
*Saccharum officinarum* L. cv. RB966928	0.1 μM	Root fertilization (2.0 mM)	Damaging the quantum efficiency of photosystem II and reducing pigment content	Decreases deficient symptoms	Enhancing antioxidant system and the Mn use efficiency	Oliveira et al., 2021
*Saccharum spontaneum* L.	0.1 μM	Root fertilization (2.0 mM)	Damaging the quantum efficiency of photosystem II and reducing pigment content	Decreases deficient symptom	Enhancing antioxidant system and the Mn uptake efficiency	Oliveira et al., 2022

## 2 The mechanisms for Si alleviation of Mn toxicity in plants

**Figure 1:**
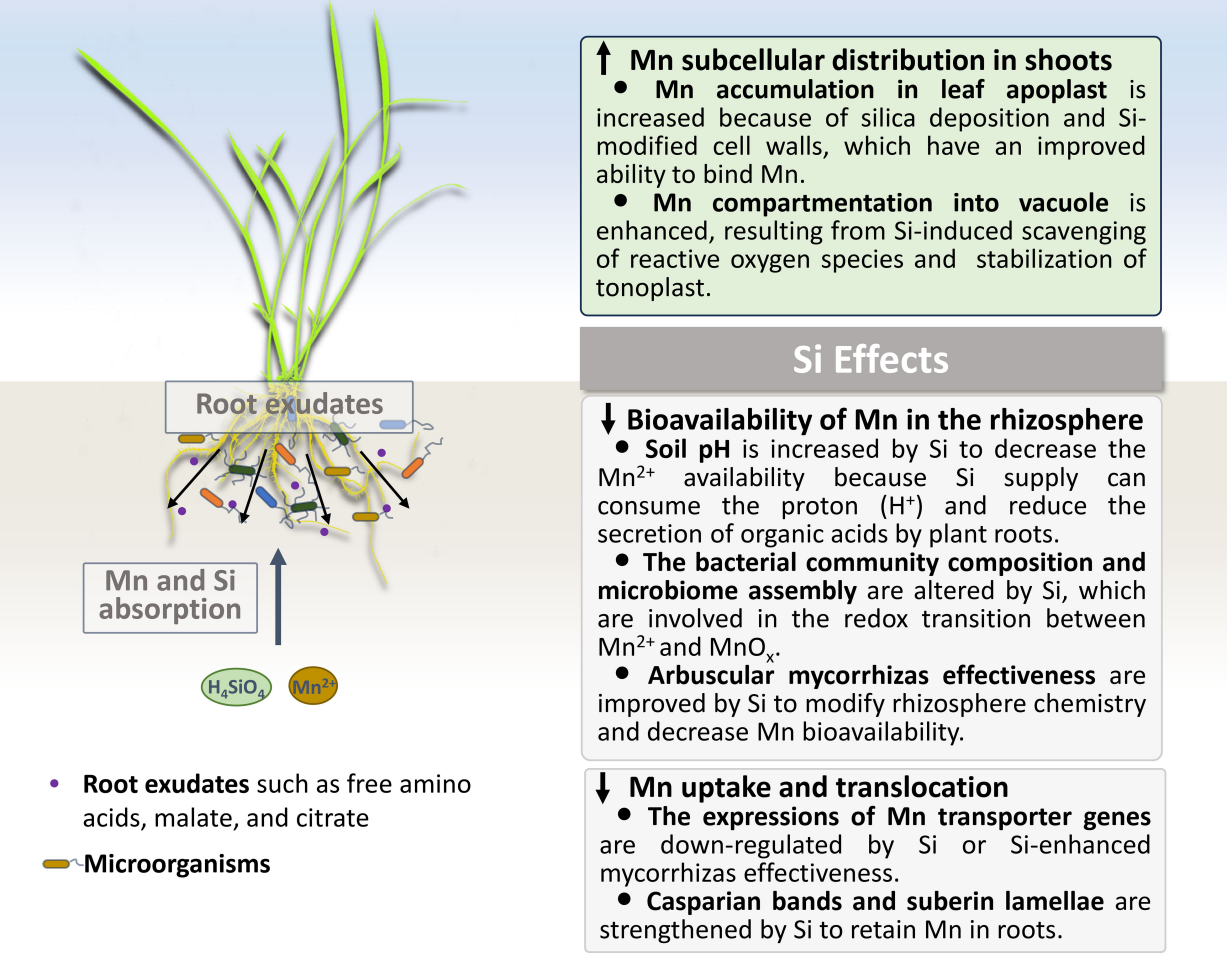
Si effects on the Mn immobilization in soil, and its uptake, translocation and subcellular compartmentation in plants under Mn-excessive conditions.

Plants grown in acidic soils may suffer from Mn toxicity, and the underlying mechanisms for the Si-reduced Mn toxicity seem to differ with plant species. In general, decreasing the bioavailability of Mn in soil, inhibiting the Mn uptake and translocation, and optimizing the distribution and allocation of Mn in plants are the main approaches to cope with Mn toxicity. Herein we will discuss the Si effects on the Mn immobilization in soil, and its uptake, translocation and subcellular compartmentation in plants (**Figure 1**).

### 2.1 Rhizosphere acidification, root exudates and microorganisms are altered by Si to decrease the bioavailability and uptake of Mn

The bioavailability of Mn in the rhizosphere mainly depends on the soil pH, root exudates, and microorganisms (Alejandro et al., 2020). In addition, the fate of Mn is closely interwoven with aluminum (Al) which is abundant in acidic soils (Shao et al., 2017). Traditional silicon fertilizers produced from wollastonite are the Lewis base, whose supply can consume the proton (H^+^) for the formation of H_4_SiO_4_ and interact with Al in the root apoplast (Hodson and Evans, 2020), leading to an increased pH of rhizosphere soil in theory. It has been reported that the uptake of Si by rice plants can significantly reduce the degree of root acidification (Ma et al., 2021; Pang et al., 2023) by inhibiting the expressions of proton pump and organic acid secretion genes (Pang et al., 2023), and the elevated soil pH decreases the Mn^2+^ availability.

Root exudation is a dynamic behavior that mediates interactions between plant roots and soil matrix. Interestingly, the leaf Mn concentrations can be used as a proxy for rhizosphere carboxylate concentrations because the root exudates mobilize the micronutrient Mn in the rhizosphere (Lambers et al., 2015, 2021; Yan et al., 2024). Under Mn-excessive conditions, root exudates of total phenolics are increased in *Citrus sinensis*, while the secretion of root total free amino acids, total soluble sugars, malate, and citrate are not altered (Zheng et al., 2024). Furthermore, a great number of researches do show that the application of Si fertilizer could reduce the secretion of organic acids by plant roots (e.g., Wu et al., 2015; Fan et al., 2018; Javed et al., 2020; Pang et al., 2023), which are observed under the normal and stress conditions. Based on these discoveries, it can be speculated that Si may immobilize the excessive micronutrient Mn by mediating the secretion of metabolites in the rhizosphere.

Moreover, root exudates have multiple effects on bacterial community composition and microbiome assembly (Vives-Peris et al., 2020; Qu et al., 2021; Li et al., 2022; Kabir et al., 2024; Shi et al., 2024), so does Si by altering root-released carboxylates and phenolics. It was proposed that Si fertilization influences microbial assemblages of rice roots in a five-season *in-situ* remediation field study (Gao et al., 2022a). In this study, Si improvement of microbial diversity and richness in the rhizosphere was detected after the third fertilization, suggesting a prolonged effect of Si fertilization on the microbiome in roots (Gao et al., 2022a). Besides working in the rhizosphere, Si enhances the abundance of reducing microbes in the rhizoplane as well (Gao et al., 2022b). Very recently, it has been reported that Si could regulate the reassembled microbial communities in soil (Gao et al., 2024).

Despite these results being involved in the Si-mediated arsenic uptake by rice plants, we still believe that Si may decrease the soil Mn^2+^ availability with microbial mechanisms. There are two reasons: (1) Mn functions as an electron donor and acceptor for microorganisms, and the anaerobic redox transition between Mn^2+^ and MnO_x_ accelerates a dynamic biogeochemical cycle coupled to microorganisms (Wang et al., 2022); (2) *Anaeromyxobacter* is closely linked to the Si-regulated microbial interactions (Gao et al., 2024), which might be involved in Mn oxidation and is capable of bioremediation in the Mn-contaminated soil (Liu et al., 2024).

In mycotrophic plants, arbuscular mycorrhizas also modify rhizosphere chemistry and influence Mn bioavailability and uptake (Brito et al., 2014; Nazeri et al., 2014). According to reports, inoculating legumes with arbuscular mycorrhizal fungi (AMF) increased the pH of the rhizosphere by around 0.2–0.7 pH units, reduced the total amount of carboxylates in the rhizosphere by 52%, and reduced the concentration of Mn in the shoots by 38% (Nazeri et al., 2014). In wheat, AMF colonization that begins with the intact extraradical mycelium improves bio-protection by decreasing Mn uptake in roots (Brito et al., 2014). Recently, it was proposed that arbuscular mycorrhizal symbiosis alleviates Mn toxicity by downregulating Mn transporter genes in *Eucalyptus tereticornis* (De Oliveira et al., 2023). Moreover, the relationships between Si and arbuscular mycorrhizas were established: AMF improve Si uptake and translocation, meanwhile Si increases mycorrhizal effectiveness in plants (Etesami et al., 2022). These results suggest an AMF-related mechanism of Si-mitigating Mn toxicity.

### 2.2 Si inhibits Mn uptake and translocation by down-regulating Mn transporter genes and reinforcing the apoplastic barriers in roots

In roots, Casparian bands intercept the apoplastic flow, resulting in the requirement of transporters for nutrient uptake and translocation. Thus, Mn uptake and translocation are governed by the expression of transporter genes and the development of apoplastic barriers. There are many Mn transporters in plants, including members of the natural resistance-associated macrophage protein (NRAMP) family, the ZRT/IRT-related protein (ZIP) family, the yellow stripe-like (YSL) family, the cation exchanger (CAX) family, the cation diffusion facilitator/metal tolerance protein (CDF/MTP) family, the vacuolar iron transporter (VIT) family and others (Shao et al., 2017; Alejandro et al., 2020).

Among the Mn transporters, OsNramp5 and OsMTP9 make up the Mn uptake system in rice roots, and OsNramp5 acts as a metal/H^+^ symporter and facilitates the Mn permeation into the root epidermis (Sasaki et al., 2012), while OsMTP9 is an efflux Mn transporter and mediates the export of Mn^2+^ into the stele (Ueno et al., 2015). Che et al. (2016) proposed that Si inhibits the Mn uptake in rice roots by down-regulating the expression of *OsNramp5* gene after a relatively long-term exposure to Si. Furthermore, the Mn concentration can be decreased in the shoots but increased in the roots due to the Si effects, suggesting that Si can reduce the root-to-shoot translocation of Mn in rice plants (Che et al., 2016). The probable mechanism for Si-reduced translocation is the formation of the Mn-Si complex in root cells.

The development of apoplastic barriers, including Casparian bands and the suberin lamellae, has a role in controlling the radial fluxes of water and nutrients and preventing the uptake of toxicants (Kreszies et al., 2020). It is widely known that Si can enhance the formation of Casparian bands and suberin lamellae by forming phenol complexes (Vaculík et al., 2012; Wu et al., 2019; Kreszies et al., 2020). Moreover, Si-modified phenols have an excellent ability to bind metals (Sheng and Chen, 2020), thereby accomplishing the Mn retention in roots. Our previous study also found that Casparian bands and Si-lignin interactions promote the silica deposition in the inner tangential cell walls of endodermis (Sheng et al., 2023). Root silica gels may serve as a pool that can store the excess Mn through the formation of the Mn-Si complex.

### 2.3 Si optimizes the subcellular distribution of Mn in shoots

In rice plants, most of the total Mn taken up by roots was translocated to the shoots, regardless of the Si presence in roots (Che et al., 2016). Additionally, Si tends to raise leaf tissue tolerance rather than induce root Mn exclusion in cucumbers (Dragišić Maksimović et al., 2007, 2012). Considering that symptoms of Mn excess mainly occur in leaves, Si alleviation of foliar Mn stress is a top priority. At the cellular level, the first strategy is that Si alters the cell wall chemistry to enhance the Mn-binding to the cell wall. For example, the lignin synthesis in cucumber leaves is altered by Si supply in response to Mn stress (Dragišić Maksimović et al., 2007), as well as the callose synthesis in maize (Doncheva et al., 2009). These results are in agreement with our assumption that Si crosslinks with different cell wall components in various plants (Sheng and Chen, 2020). In Si-accumulating plants, leaf apoplast is one of the main locations for silica deposition, relevant or irrelevant to the cell wall (Hodson, 2019). It is likely that the Mn-Si complex can form in the leaf apoplast of Si-accumulating plants to prevent the uptake of Mn into the cytoplasm. Taken together, cell wall-bound Si and silica mediate the Mn accumulation in leaf apoplast for reducing the Mn toxicity.

Once the excess Mn^2+^ ions enter the cytoplasm, the intracellular reactions will be triggered. To avoid metal toxicity, Mn compartmentation into vacuole is one of the most important ways (Sharma et al., 2016; He et al., 2021), a process which depends on the tonoplast stability and tonoplast-localized transporter. To our knowledge, there is no evidence to show that some tonoplast-localized Mn transporters are regulated by Si. Furthermore, Si significantly strengthens the capacity of antioxidant system to scavenge reactive oxygen species (ROS), which can harm the membrane system. It means that Si increases the stability of membrane system, including the tonoplast, under stress conditions (Sheng et al., 2024). This, in turn, enhances the activity of tonoplast-localized Mn transporters (Sheng et al., 2018, 2024). Thus, Si has the potential ability to mediate the Mn compartmentation into the vacuole, but this should still be elucidated. Similar results have been reported in Cd-stressed rice cells: In the case of Cd, Si addition can reduce the Cd toxicity by compartmentation of Cd into vacuoles (Ma et al., 2016). Overall, it can be concluded that Si optimizes the subcellular distribution of Mn in shoots.

## 3 The roles of Si in ameliorating Mn deficiency

Mn deficiency often occurs in plants that grow in alkaline, well-aerated, and calcareous soils (Alejandro et al., 2020). In such soils, plant-available Mn^2+^ is readily oxidized and then converted to insoluble Mn oxides (MnO_x_). When sorghum and energy cane (*Saccharum spontaneum* L.) plants are exposed to Mn deficient environment, the Si fertilization of the root results in an increase in Mn use efficiency (Oliveira et al., 2021, 2022). Owing to a lack of investigations about the Si effects on Mn availability in the soil and the expression of Mn transporters, there is no way to know the underlying mechanisms for Mn uptake in sorghum and energy cane. In cucumber plant, Si application can prevent certain symptoms of Mn deficiency without any effects on the Mn uptake and accumulation (Bityutskii et al., 2014), suggesting a different mechanism in addition to Si enhancement of Mn uptake. It can be explained by Si-reducing the accumulation of ROS in plant tissues, which is a common mechanism among plant species and will be discussed in the next section.

## 4 Scavenging of reactive oxygen species is a common mechanism for Si alleviation of Mn disorder

The ROS burst is one of the most common phenomena in different cell compartments, resulting from the environment stress-induced disruption of cellular homeostasis. Both Mn deficiency and excess can trigger the ROS burst and then damage organelle membranes (Li et al., 2012; Oliveira et al., 2021, 2022). Many investigations indicate that Si mediates Mn disorder by scavenging reactive oxygen species in plants (Dragišić Maksimović et al., 2012; Oliveira et al., 2021, 2022). However, the effects of Si on the antioxidant defense system (including enzymatic and non-enzymatic antioxidants) differ from plant species under Mn excess (Dragišić Maksimović et al., 2007, 2012; Li et al., 2012). For instance, Si supply decreases the hydroxyl radical accumulation and suppresses the Mn-induced increased activity of peroxidase (POD) isoforms in cucumber (Dragišić Maksimović et al., 2012); while Si significantly counteracts high Mn-elevated malondialdehyde (MDA) and H_2_O_2_ concentrations and suppresses the Mn-induced increased activity of superoxide dismutase (SOD), catalase (CAT) and ascorbate peroxidase (APX) in Mn-sensitive rice plants (Li et al., 2012). Moreover, glutathione (GSH), non-protein thiols (NPT), and ascorbic acid (AsA) concentrations in rice are increased after Si addition (Li et al., 2012), as well as chlorogenic acid and caffeic acid in cucumber (Dragišić Maksimović et al., 2007). These results suggest that Si mainly influences non-enzymatic rather than enzymatic antioxidants in plants under high Mn stress.

Under Mn deficiency, Si reduces H_2_O_2_ and MDA contents and increases the SOD activity, phenol contents, thus improving the growth of energy cane (Oliveira et al., 2021, 2022). Similar results were also reported in sorghum plants, implying that Si enhances both enzymatic and non-enzymatic antioxidants (de Oliveira et al., 2019). In any case, Si accelerates the degradation of ROS and prevents the peroxidation of membrane systems under both Mn deficient and excessive conditions, with roles in attenuating the symptoms induced by Mn disorder and increasing photosynthetic activity in different plants (Li et al., 2015; de Oliveira et al., 2019).

## 5 Conclusions and future perspectives

Si regulates the Mn homeostasis in many plants with varying mechanisms. In sum, there are three approaches for Si to alleviate Mn stress: reducing the bioavailability of Mn in the rhizosphere, inhibiting Mn uptake and translocation, and optimizing the subcellular distribution of Mn in shoots. Furthermore, scavenging of reactive oxygen species is a common mechanism for Si alleviation of Mn disorder. Unfortunately, how Si promotes Mn uptake and accumulation in Mn-deficient plants is still unclear and the mechanisms for Si-attenuating high Mn stress are not fully known. To address these questions, the Si-Mn interactions with plant cell walls, ROS, and rhizosphere microorganisms should be considered in the future. If the Si-Mn interactions with plant cell walls are investigated in detail, how Si regulates the expression of Mn transporter genes will be addressed. Because the alterations of cell wall by Si, Mn, or both can be sensed by cell wall integrity sensors such as wall-associated kinases (WAKs) and FERONIA kinase family members (Wolf, 2022), which will trigger the cell wall integrity signaling and then reprogram the transcriptome of plants. Overall, these efforts will help improve our understanding of the Si roles in regulating Mn homeostasis.
